# Characteristics of Associated Injuries in Children and Teenagers With Craniofacial Fractures

**DOI:** 10.1097/SCS.0000000000009343

**Published:** 2023-05-19

**Authors:** Aura Kirvelä, Johanna Snäll, Auli Suominen, Tero Puolakkainen, Hanna Thorén

**Affiliations:** * Department of Oral and Maxillofacial Surgery, Institute of Dentistry, University of Turku, Turku, Finland; † Department of Oral and Maxillofacial Diseases, Turku University Hospital, Turku, Finland; ‡ Department of Oral and Maxillofacial Diseases, University of Helsinki and Helsinki University Hospital, Helsinki, Finland; § Department of Community Dentistry, University of Turku, Turku, Finland

**Keywords:** Children, comorbidities, comorbidity, maxillofacial injuries, maxillofacial injury, teenager

## Abstract

The incidence of pediatric craniofacial fractures and heterogeneity of fractures is known to increase with age. This study aimed to determine the occurrence of associated injuries (AIs) to craniofacial fractures and identify differences in patterns of and predictors for AIs in children and teenagers. A 6-year retrospective cross-sectional cohort study was designed and implemented. The study population included 397 patients aged 19 years or less diagnosed with craniofacial fracture at Helsinki University Hospital from 2013 to 2018. Boys (71.0%) and teenagers (64.7%) were predominated. Associated injuries were more common in teenagers than children. Teenagers had more often AI in 2 or more organ systems. Assault and intoxication by alcohol were observed only in teenagers and predominantly boys. A total of 27.0% of all patients sustained AIs. In 18.1%, brain injury was reported. In children, motor vehicle accident (MVA) was an independent predictor for AI. In teenagers, independent predictors for AI were female sex, isolated cranial fracture, combined cranial fracture, and high-energy trauma mechanism. Injury patterns and AI related to craniofacial fractures in the pediatric population are age-specific, requiring multidisciplinary collaboration in the diagnosis, treatment, and follow-up of such trauma. Predictors for AIs increase in complexity with age, and the role of sex as a predictor is evident in teenagers.

Craniofacial fractures in children are rare in comparison with adults. Small children seldom sustain facial fractures due to a protected lifestyle and specific anatomic characteristics of the maturing facial skeleton and cranium. According to previous studies on pediatric facial fractures, some 15% of facial fractures occur in children.^1–3^ There is evidence of a higher incidence of facial fractures with increasing age within the pediatric population.^1,4^ Children’s facial fractures tend to affect the mandible, though there are indications of increased fracture site heterogeneity with increasing age.^5^

Previous studies on pediatric facial fractures have reported brain injuries and other associated injuries (AI).^6–8^ This is reflected in costly and lengthy treatment, as pediatric facial fractures are often associated with polytrauma.^3,9^ Data on specific patterns of AI related to craniofacial fractures in different age groups are scarce.

The present study aimed to investigate trauma mechanisms and injury patterns in children and teenagers with fractures of the craniofacial skeleton. The specific aims were to determine the occurrence of AIs and identify differences in patterns of and predictors for AIs in children and teenagers. The hypothesis was that there were significant differences between the age groups.

## METHODS

A retrospective, cross-sectional cohort study was designed and implemented to address the research aims. Data were collected from all patients diagnosed with any craniofacial fracture at Helsinki University Hospital between 2013 and 2018 and evaluated retrospectively. This study included all patients who were aged 19 years or less at the time of injury.

### Study Variables

The primary outcome variable was AI, defined as brain injury (verified by magnetic resonance imaging or computed tomography), neck injury (cervical spine injuries and blunt cerebrovascular injuries), spinal injury (injury to the thoracic and lumbosacral spine), chest injury (thoracic injury), abdominal injury, pelvic injury, and limb injury (joint dislocations and fractures of the upper and lower extremities). Brain concussion, wounds, and other minor soft tissue injuries were not included. The secondary outcome variables were affected organ systems and ≥2 affected organ systems. The affected organ system was classified as brain, neck, spine, chest, abdomen, pelvis, and limb.

The primary predictor variable was the age group (children and teenagers). In this study, all patients up to 12 years old were classified as children, and patients between 13 and 19 years old as teenagers. Secondary predictor variables were sex, type of craniofacial fracture, etiology, high-energy trauma mechanism, assault, and intoxication by alcohol.

Type of craniofacial fracture was classified as isolated facial fracture (including the mandible and/or midface), isolated cranial fracture (including the frontal bone, orbital roof, or other parts of the skull vault or skull base), and combined facial and cranial fracture. Etiology was classified into 6 categories: hit by fist or kicked, fall from height or in stairs, fall on ground level, motor vehicle accident (MVA), nonmotorized means of transport (ie, bicycle and sledding accidents), and hit by an object. Also, the need for hospitalization and intensive care, and mortality during hospitalization were recorded. Motor vehicle accidents and falls from heights or stairs were defined as high-energy trauma mechanisms.

### Statistical Analysis

The Pearson χ^2^ test was used to examine the association between secondary predictors and primary predictors. The odds ratio (OR) was calculated to determine the difference between age groups in terms of AI. Relative risks were calculated, processing statistical significances with Pearson χ^2^ test to assess the relationship between predictors and outcome variables in both age groups. When more than 1 statistically significant predictor with risk ratio (RR) >2 was identified, multivariable logistic regression analysis was conducted to identify independent predictors as OR. If the model estimation calculation was unsuccessful, nonestimated covariables were excluded, and the likelihood estimation was repeated. *P*<0.05 was considered as the threshold for statistical significance. Statistical analysis was performed using SAS statistical software 9.4 (SAS Institute Inc., Cary, NC).

### Ethical Considerations

The study was approved by the Internal Review Board of the Head and Neck Center, Helsinki University Hospital, Helsinki, Finland (HUS/356/2017).

## RESULTS

A total of 397 children and teenagers were included in the study. Supplemental Table 1, Supplemental Digital Content 1, http://links.lww.com/SCS/E948 shows the characteristics of these patients. Boys (71.0%) and teenagers (64.7%) were predominated. The commonest mechanisms of injury were hits by fists or kicks (20.2%) and fall from height or stairs (19.6%). High-energy trauma mechanisms (34.3%) were frequent. A total of 14.6% of the patients were under the influence of alcohol at the time of the injury. Cranial fractures occurred in 36.5%, more often in isolation (25.2%) than in combination with facial fractures (11.3%). AI occurred in 27.0%, most often in only 1 organ system (16.4%). The brain was most often affected (18.1%). Hospitalization was required for 69.5%, most often for only 1 day (24.9%). Treatment in intensive care was required for 13.9%. The mortality rate was 1.8% for the entire study population.

Supplemental Table 2, Supplemental Digital Content 1, http://links.lww.com/SCS/E948 shows the association between variables and age group. There were significant differences between children and teenagers. Boys represented a majority in both age groups, and their proportion was even larger in teenagers (*P*=0.001). A high-energy trauma mechanism in general (*P*=0.001), specifically falls from height or in stairs (*P*<0.001), was significantly more common in children than in teenagers, as were accidents caused by nonmotorized means of transport (*P*=0.003). Motor vehicle accidents were more common in teenagers than in children (*P*=0.001). Assault and intoxication by alcohol were observed only in teenagers. The great majority of intoxicated patients (50 of 58) and patients involved in assault (69 of 75) were boys. Mortalities were observed only among teenagers (2.7%). Isolated facial fractures were more common in teenagers (*P*<0.001), whereas isolated cranial fractures were more common in children (*P*<0.001). Limb injuries were more common in teenagers (*P*=0.006). Teenagers had more often AI in 2 or more organ systems (*P*=0.047).

Supplemental Table 3, Supplemental Digital Content 1, http://links.lww.com/SCS/E948 summarizes the multivariable regression analysis for AI. Teenagers had 2.17 times greater odds for AI than children when adjusted for sex, etiology, high-energy mechanism, and assault. Teenagers also had 3.29 greater odds for limb injuries than children.

Supplemental Table 4, Supplemental Digital Content 1, http://links.lww.com/SCS/E948 shows the significant predictors for outcome variables for children. Children had fewer significant predictors than teenagers. Motor vehicle accident (OR=7.14) was an independent predictor for AI. Isolated cranial fracture (OR=3.10) and MVA (OR=4.92) were independent predictors for brain injury. Being hit by an object was an independent predictor for neck injuries (RR=32.76), chest injuries (RR=7.25), and ≥2 AI sites (RR=3.87). Male sex was an independent predictor for spinal injuries (RR=3.35). The MVA was an independent predictor for limb injuries (RR=14.56).

Supplemental Table 5, Supplemental Digital Content 1, http://links.lww.com/SCS/E948 shows the significant predictors for outcome variables for teenagers. Independent predictors for AI were isolated cranial fractures (OR=37.56), combined facial and cranial fractures (OR=7.02), and high-energy trauma mechanism (OR=5.96). Independent predictors for brain injury were isolated cranial fracture (OR=37.56), combined facial and cranial fracture (OR=13.49), and MVA (OR=5.96). Independent predictors for spinal injury were isolated cranial fracture (OR=6.83) and high-energy trauma mechanism (OR=23.00). Independent predictors for chest injury were combined facial and cranial fracture (OR=16.33) and fall from height or stairs (OR=10.41). Independent predictors for abdominal injury were combined facial and cranial fracture (OR=8.66) and fall from height or stairs (OR=7.31). High-energy trauma mechanism (OR=11.64) was an independent predictor for limb injury. Independent predictors for ≥2 AI sites were isolated cranial fracture (OR=4.47), combined facial and cranial fracture (OR=5.20), and high-energy trauma mechanism (OR=15.78).

Sex was found to be a significant predictor for AI and certain AI subtypes. In children, boys were more likely to have spinal injuries (RR=3.35). In teenagers, girls were more likely to have AI (OR=3.47), neck injuries (RR=13.42), pelvic injuries (RR=4.20), and limb injuries (RR=2.47).

## DISCUSSION

The present study aimed to investigate trauma mechanisms and injury patterns in children and teenagers with fractures of the craniofacial skeleton. The specific aims were to determine the occurrence of AIs in general and possible differences in patterns of AIs between children and teenagers.

Children and teenagers differed regarding etiology and sustained injuries. In children, high-energy trauma mechanisms, particularly falls from heights or stairs were common. Assault and intoxication by alcohol were frequent in teenagers and occurred almost exclusively among boys.

An isolated facial fracture was the most common overall fracture type, occurring in 63.5% of all patients. However, isolated cranial fractures were significantly more frequent among children than among teenagers. The finding is at least partly explained by age-related differences in the anatomic relationship between the face and skull. The skull is large, and the frontal bone is prominent in children and therefore susceptible to injury. As a result of forward and downward growth of the mandible and midface, the facial skeleton becomes more susceptible to fracture with increasing age.

Some predictors were shared by both age groups. Predictors for AI for both age groups were combined facial and cranial fracture, MVA, and high-energy trauma mechanisms. For brain injury, shared predictors were isolated cranial fracture and MVA. Motor vehicle accident was a predictor for limb injury in both age groups. These findings indicate that when the severity of MVA is such that it results in cranial fractures, AI, specifically brain injury, is often present regardless of age group. Predictors for other injury types differed, and more predictors per injury type were identified for teenagers than children. For children, the most common predictors across AI types were being hit by an object and MVA, whereas for teenagers, they were falls from height and in stairs, MVA, and high-energy trauma mechanism. Teenage girls were found to have a pronounced incidence of neck, pelvic, and limb injuries. There is evidence of adult females being more susceptible to whiplash injuries than men, thought to be explained by anatomic differences in neck musculature and body weight distribution.^10^ The results of this study are in line with earlier findings, as neck injuries in teenagers were associated with female sex, MVA, and high-energy trauma mechanism. Women generally have weaker upper body and neck musculature, and their body mass tends to be distributed more around the hip area than in men. Falls from height and body mass concentration around the lower body may in part explain the higher risk of pelvic and limb injuries in teenage girls. In teenagers, high-energy trauma mechanisms were predictors for AI and all AI subtypes. Cranial fractures, either isolated or in combination with facial fractures, were also common predictors for AI and all AI types but neck injury in teenagers. Teenagers had several predictors for all AI subtypes. Together, these findings speak of a more heterogenous and severe nature of the circumstances leading to craniofacial fractures with increasing age. To address the prevention of craniofacial fractures and subsequent AI, age, and sex should be considered.

AI occurred in 27.0%, brain injuries being the most common (18.1%). In children 12 years and under, we observed AI in 25.0%. In an earlier study of children under 15 years of age with mandibular and/or mid-facial fractures, the rate of AI was 11.0%.^11^ The difference in patient material explains the difference in the rate of AI; the present study also included patients with cranial fractures, which were highly frequently (69.2%) associated with high-energy trauma mechanisms. Moreover, the overall rate of high-energy trauma mechanisms was much higher in the children in our study (45.0%) than in the aforementioned study (28.6%), predisposing them to AIs. However, the total mortality rate was low (1.8%), which has been observed previously, too (3.3%).^12^

In previously published investigations, the rates of traumatic brain injuries in young patients with facial fractures have varied greatly, ranging between 11.5% and 41.9%.^6,8,13–15^ The 18.1% rate of brain injuries in the present study sets at the lower end of what has been reported previously. One reason is that we only included brain injuries that had been verified with computed tomography or magnetic resonance imaging. Diagnosing brain injury is challenging and controversial. Had concussions been included in this study, the rate of brain injury would have been higher. As shown by a study that utilized the Finnish National Hospital Discharge Register, concussions were by far the most common traumatic brain injuries in patients aged 18 years or less who had been hospitalized for traumatic brain injuries from 1998 to 2012.^16^ It is important to acknowledge that between 14% and 29% of children with mild traumatic brain injury show postconcussion symptoms for several months after the injury.^17^ Mild traumatic brain injuries need to be identified, and referral to rehabilitation is mandatory if symptoms persist.^17^

Interpersonal violence as etiology (38–43%)^18,19^ and being intoxicated by alcohol (31%–55%)^20,21^ are common findings in adult patients with facial fractures. An unfortunate finding in the present study was the high frequency of combined assault and intoxication by alcohol among teenage boys. Earlier studies have likewise reported assault as the most common etiology in patients aged 18 years or less, particularly among boys.^6,15^ Screening of alcohol use must be performed routinely whenever an intoxicated child is encountered, and appropriate intervention should be available. Traumatologists should take these aspects into account when examining teenage patients with facial fractures.

Limitations of this study include its retrospective nature. As patient data are not documented in a structured form, it was not possible to confirm the absence or presence of all interesting variables. For example, the rate of intoxication by alcohol may therefore be underestimated. The period of data collection provides extensive enough patient material for meaningful and reliable statistical analysis.

## CONCLUSIONS

This study found significant differences in injury patterns and AI between children and teenagers with craniofacial fractures. Associated injuries in children are severe when they occur, mainly in the form of brain injury secondary to cranial fractures. In teenage facial fracture patients, the focus should be on identifying assaults. Assault-related injuries and intoxication are common in teenagers, especially in boys, calling for active screening for alcohol use and appropriate intervention when needed. Predictors for AI differ between children and teenagers, with teenagers exhibiting a more diverse etiological background. In teenagers, with increasing age, sex, and related anatomic differences are reflected in etiological patterns seen in adult populations.

## Supplementary Material
