# Supplementary material for: Characteristics of Associated Injuries in Children and Teenagers With Craniofacial Fractures
Source: J Craniofac Surg. 2023 May 19;34(6):1625–8. doi: 10.1097/SCS.0000000000009343 (PMC10445634; doi:10.1097/SCS.0000000000009343)
Supplement: Supplementary file 1 [file scs-34-1625-s001.docx]

| **Supplemental Table 1. Characteristics of 397 children and teenagers with craniofacial fractures** | | |  |
| --- | --- | --- | --- |
|  |  | **n** | **%** |
|  |  |  |  |
| **Gender** | Male | 282 | 71.0 |
|  | Female | 115 | 29.0 |
| **Age (years)** | Range | 0-19 |  |
|  | Average | 13.4 |  |
| **Age group** | Children | 140 | 35.3 |
|  | Teenagers | 257 | 64.7 |
| **Etiology** | Hit by fist/kicked | 80 | 20.2 |
|  | Fall from height/in stairs | 78 | 19.6 |
|  | Hit by object | 71 | 17.9 |
|  | Non-motorized mean of transport | 64 | 16.1 |
|  | Motor vehicle accident | 58 | 14.6 |
|  | Fall on ground level | 43 | 10.8 |
|  | Unknown | 3 | 0.8 |
| **High-energy trauma mechanism** | Yes | 136 | 34.3 |
| **Assault** | Yes | 74 | 18.6 |
| **Alcohol** | Yes | 58 | 14.6 |
| **Type of craniofacial fracture** | Isolated facial | 252 | 63.5 |
|  | Isolated cranial | 100 | 25.2 |
|  | Combined | 45 | 11.3 |
| **AI present** | Yes | 107 | 27.0 |
| **AI per site** | Brain injury | 72 | 18.1 |
|  | Limb injury | 39 | 9.8 |
|  | Chest injury | 23 | 5.8 |
|  | Spinal injury | 16 | 4.0 |
|  | Abdominal injury | 10 | 2.5 |
|  | Pelvic injury | 9 | 2.3 |
|  | Neck injury | 8 | 2.0 |
|  | AI in ≥ 2 sites | 42 | 10.6 |
| **Hospitalization** | Yes | 276 | 69.5 |
| **Intensive care** | Yes | 55 | 13.9 |
| **Mortality** | Yes | 7 | 1.8 |
| Abbreviation: AI, associated injury |  |  |  |

| **Supplemental Table 2. Association between secondary and primary predictors** | | | |  |  |
| --- | --- | --- | --- | --- | --- |
|  |  |  |  |  |  |
|  | **Children (n=140)** | | **Teenagers (n=257)** | |  |
|  | **n** | **% of 140** | **n** | **% of 257** | **P value** |
|  |  |  |  |  |  |
| **Gender** |  |  |  |  | 0.001 |
| Male | 84 | 60 | 198 | 77 |  |
| Female | 56 | 40 | 59 | 23 |  |
|  |  |  |  |  |  |
| **Etiology** |  |  |  |  |  |
| Hit by fist/kicked | 0 | 0 | 80 | 31 | NA |
| Fall from height/in stairs | 54 | 39 | 24 | 9 | <0.001 |
| Hit by object | 24 | 17 | 47 | 18 | >0.05 |
| Non-motorized mean of transport | 33 | 24 | 31 | 12 | 0.003 |
| Motor vehicle accident | 9 | 6 | 49 | 19 | 0.001 |
| Fall on ground level | 18 | 13 | 25 | 10 | >0.05 |
| Unknown | 2 | 1 | 1 | 0 | >0.05 |
|  |  |  |  |  |  |
| **Type of craniofacial fracture** |  |  |  |  |  |
| Isolated facial | 58 | 41 | 194 | 75 | <0.001 |
| Isolated cranial | 72 | 51 | 28 | 11 | <0.001 |
| Combined facial and cranial fracture | 10 | 7 | 35 | 14 | >0.05 |
|  |  |  |  |  |  |
| **AI present** | 35 | 25 | 72 | 28 | >.05 |
| **AI in ≥ 2 affected sites** | 9 | 6 | 33 | 13 | 0.047 |
|  |  |  |  |  |  |
| **AI per site** |  |  |  |  |  |
| **Brain injury** | 28 | 20 | 44 | 17 | >.05 |
| **Neck injury** | 3 | 2 | 5 | 2 | >.05 |
| **Spinal injury** | 2 | 1 | 14 | 5 | >.05 |
| **Chest injury** | 5 | 4 | 18 | 7 | >.05 |
| **Abdominal injury** | 3 | 2 | 7 | 3 | >.05 |
| **Pelvic injury** | 0 | 0 | 9 | 4 | NA |
| **Limb injury** | 6 | 4 | 33 | 13 | 0.006 |
|  |  |  |  |  |  |
| **Hospitalization** | 102 | 73 | 174 | 68 | >.05 |
| **Intensive care** | 13 | 9 | 42 | 16 | >.05 |
| **Mortality** | 0 | 0 | 7 | 3 | NA |
|  |  |  |  |  |  |
| **High-energy trauma mechanism** | 63 | 45 | 73 | 28 | 0.001 |
| **Assault** | 0 | 0 | 74 | 29 | NA |
| **Alcohol** | 0 | 0 | 58 | 23 | NA |
| Abbreviation: AI, associated injury |  |  |  |  |  |

| **Supplemental Table 3. Logistic regression analysis between outcome variables and age group** | | | |  |
| --- | --- | --- | --- | --- |
|  |  |  |  |  |
|  | **OR** | |  |  |
|  | **Teenagers** | **Children** | **95% CI** | **P value** |
| **AI** | 2.17^b^ | 1.0 | 1.21-3.92 | 0.01 |
| **Brain injury** | 1.0 | 1.21 | 0.72-2.05 | 0.477 |
| **Neck injury** | 1.0 | 1.10 | 0.26-4.69 | 0.894 |
| **Spinal injury** | 3.98 | 1.0 | 0.89-1.75 | 0.071 |
| **Chest injury** | 2.03 | 1.0 | 0.74-5.60 | 0.17 |
| **Abdominal injury** | 1.28 | 1.0 | 0.33-5.02 | 0.725 |
| **Pelvic injury** | NA | NA | NA | NA |
| **Limb injury** | 3.29 | 1.0 | 1.34-8.06 | 0.009 |
| **AI in ≥ 2 sites** | 2.11 | 1.0 | 1.0-4.62 | 0.052 |
| ^a^ adjusted for gender, etiology, high-energy mechanism, assault, intoxication | | | |  |
| ^b^ adjusted for gender, etiology, high-energy mechanism, assault | | | |  |
| Abbreviation: AI, associated injury | | |  |  |

| **Supplemental Table 4. Significant predictors for the presence of AI and AI subtypes in children** | | | | |
| --- | --- | --- | --- | --- |
|  |  |  |  |  |
| **Predictors for** | **RR** | **P value** | **OR** | **P value** |
| **AI:** |  |  |  |  |
| Combined facial and cranial fracture | 2.17 (1.08 - 4.34) | 0.0292 |  |  |
| Motor vehicle accident* | 3.64 (2.25 - 5.88) | <0.0001 | 7.14 (1.30 - 39.19) | 0.0236 |
| High energy trauma mechanism | 2.32 (1.27 - 4.33) | 0.0065 |  |  |
| **Brain injury:** |  |  |  |  |
| Isolated cranial fracture* | 2.83 (1.29 - 6.23) | 0.0096 | 3.10 (1.09 - 8.79) | 0.0332 |
| Motor vehicle accident* | 3.16 (1.58 - 6.31) | 0.0011 | 4.92 (1.05 - 23.00) | 0.0427 |
| High energy trauma mechanism | 2.58 (1.26 - 5.30) | 0.0099 |  |  |
| **Neck injury:** |  |  |  |  |
| Hit by object* | 32.76 (1.75 - 614.50) | 0.0197 |  |  |
| **Spinal injury:** |  |  |  |  |
| Male* | 3.35 (0.16 - 68.56) | 0.0432 |  |  |
| **Chest injury:** |  |  |  |  |
| Hit by object* | 7.25 (1.28 - 41.08) | 0.0252 |  |  |
| **Limb injury:** |  |  |  |  |
| Motor vehicle accident* | 14.56 (2.31 - 91.65) | 0.0043 |  |  |
| **AI in ≥ 2 sites:** |  |  |  |  |
| Hit by object* | 3.87 (1.12 - 13.35) | 0.0324 |  |  |
| *Independent predictor |  |  |  |  |
| Abbreviation: AI, associated injury |  |  |  |  |

| **Supplemental Table 5. Significant predictors for the presence of AI and AI subtypes in teenagers** | | | | |
| --- | --- | --- | --- | --- |
|  |  |  |  |  |
| **Predictors** | **RR** | **P value** | **OR** | **P value** |
| **AI** |  |  |  |  |
| Female* | 1.90 (1.29 - 2.78) | 0.0011 | 3.47 (1.26 - 9.52) | 0.0152 |
| Isolated cranial* | 3.37 (2.44 - 4.65) | <0.0001 | 19.42 (6.14 - 61.50) | <0.0001 |
| Combined facial and cranial fracture* | 3.17 (2.27 - 4.44) | <0.0001 | 7.02 (2.44 - 20.16) | 0.0003 |
| Fall from height/in stairs | 2.55 (1.74 - 3.75) | <0.0001 |  |  |
| Motor vehicle accident | 5.02 (3.56 - 7.07) | <0.0001 |  |  |
| High-energy trauma mechanism* | 7.56 (4.78 - 11.97) | <0.0001 | 29.23 (11.24 - 76.04) | <0.0001 |
| **Brain injury** |  |  |  |  |
| Isolated cranial fracture* | 5.89 (3.71 - 9.34) | <0.0001 | 37.56 (11.96 - 117.95) | <0.0001 |
| Combined facial and cranial fracture* | 4.15 (2.52 - 6.82) | <0.0001 | 13.49 (4.66 - 39.06) | <0.0001 |
| Motor vehicle accident* | 5.90 (3.51 - 9.92) | <0.0001 | 5.96 (1.45 - 24.29) | 0.0132 |
| High-energy trauma mechanism | 6.01 (3.34 - 10.82) | <0.0001 |  |  |
| **Neck injury** |  |  |  |  |
| Female | 13.42 (1.53 - 117.80) | 0.0191 |  |  |
| Motor vehicle accident | 6.37 (1.09 - 37.08) | 0.0395 |  |  |
| High-energy trauma mechanism | 10.08 (1.15 - 88.70) | 0.0373 |  |  |
| **Spinal injury** |  |  |  |  |
| Isolated cranial fracture* | 8.18 (3.10 - 21.61) | <0.0001 | 6.83 (1.91 - 24.45) | 0.0032 |
| Fall from height/in stairs | 7.28 (2.76 - 19.24) | 0.0001 |  |  |
| Motor vehicle accident | 4.25 (1.56 - 11.54) | 0.0046 |  |  |
| High-energy trauma mechanism* | 32.77 (4.37 - 245.98) | 0.0007 | 23.00 (2.67 - 197.83) | 0.0043 |
| **Chest injury** |  |  |  |  |
| Combined facial and cranial fracture* | 9.97 (4.14 - 23.98) | <0.0001 | 16.33 (5.16 - 51.68) | <0.0001 |
| Fall from height/in stairs* | 6.18 (2.64 - 14.45) | <0.0001 | 10.41 (2.89 - 37.70) | 0.0013 |
| Motor vehicle accident | 6.67 (2.73 - 16.33) | <0.0001 |  |  |
| High-energy trauma mechanism | 92.50 (5.65 - 1515.21) | 0.0015 |  |  |
| **Abdominal injury** |  |  |  |  |
| Combined facial and cranial fracture* | 8.46 (1.98 - 36.20) | 0.0040 | 8.66 (1.76 - 42.45) | 0.0078 |
| Fall from height/in stairs* | 7.28 (1.73 - 30.63) | 0.0068 | 7.31 (1.41 - 37.83) | 0.0177 |
| Motor vehicle accident | 5.66 (1.31 - 24.48) | 0.0203 |  |  |
| High-energy trauma mechanism | 37.50 (2.17 - 648.32) | 0.0127 |  |  |
| **Pelvic injury** |  |  |  |  |
| Female | 4.20 (1.16 - 15.12) | 0.0284 |  |  |
| Combined facial and cranial fracture | 5.07 (1.43 - 17.99) | 0.0119 |  |  |
| Fall from height/in stairs | 7.77 (2.23 - 27.00) | 0.0013 |  |  |
| Motor vehicle accident | 5.31 (1.48 - 19.04) | 0.0105 |  |  |
| High-energy trauma mechanism | 47.50 (2.80 - 805.76) | 0.0075 |  |  |
| **Limb injury** |  |  |  |  |
| Female* | 2.47 (1.32 - 4.63) | 0.0046 | 2.54 (1.07 - 6.05) | 0.0348 |
| Combined facial and cranial fracture | 3.17 (1.69 - 5.95) | 0.0003 |  |  |
| Fall from height/in stairs | 3.11 (1.58 - 6.11) | 0.001 |  |  |
| Motor vehicle accident | 5.09 (2.77 - 9.38) | <0.0001 |  |  |
| High-energy trauma mechanism* | 9.36 (4.25 - 20.62) | <0.0001 | 11.64 (4.27 - 31.75) | <0.0001 |
| **AI in ≥ 2 sites** |  |  |  |  |
| Isolated cranial fracture* | 3.07 (1.59 - 5.92) | 0.0008 | 4.47 (1.39 - 14.33) | 0.0118 |
| Combined facial and cranial fracture* | 4.67 (2.59 - 8.44) | <0.0001 | 5.20 (1.78 - 15.19) | 0.0026 |
| Fall from height/in stairs | 4.22 (2.29 - 7.79) | <0.0001 |  |  |
| High-energy trauma mechanism* | 18.27 (6.66 - 50.16) | <0.0001 | 15.78 (4.74 - 52.60) | <0.0001 |
| *Independent predictor |  |  |  |  |
| Abbreviation: AI, associated injury |  |  |  |  |
